# COVID‐19‐associated Brugada pattern electrocardiogram: Systematic review of case reports

**DOI:** 10.1111/anec.13051

**Published:** 2023-02-21

**Authors:** Kevin Liu, Kai Chen, Ekin C. Uzunoglu, Azeem Rathore, Tanya Deol, Ele Wu, Claude S. Elayi, Prakash G. Suryanarayana, Stephen G. Keim, John N. Catanzaro

**Affiliations:** ^1^ Department of Medicine, Division of Cardiology, Section of Electrophysiology University of Florida Health Science Center Florida Jacksonville USA; ^2^ CHI Saint Joseph Hospital – Cardiology Lexington Kentucky USA

**Keywords:** Brugada, COVID, electrocardiogram

## Abstract

**Aims:**

To summarize published case reports of patients diagnosed with coronavirus disease 2019 (COVID‐19) and Brugada pattern electrocardiogram (ECG).

**Methods:**

The Preferred Reporting Items for Systematic Reviews and Meta‐Analyses checklist were followed. A literature search was conducted using PubMed, EMBASE, and Scopus up until September 2021. The incidence, clinical characteristics, and management outcomes of COVID‐19 patients with a Brugada pattern ECG were identified.

**Results:**

A total of 18 cases were collected. The mean age was 47.1 years and 11.1% were women. No patients had prior confirmed diagnosis of Brugada syndrome. The most common presenting clinical symptoms were fever (83.3%), chest pain (38.8%), shortness of breath (38.8%), and syncope (16.6%). All 18 patients presented with type 1 Brugada pattern ECG. Four patients (22.2%) underwent left heart catheterization, and none demonstrated the presence of obstructive coronary disease. The most common reported therapies included antipyretics (55.5%), hydroxychloroquine (27.7%), and antibiotics (16.6%). One patient (5.5%) died during hospitalization. Three patients (16.6%) who presented with syncope received either an implantable cardioverter defibrillator or wearable cardioverter defibrillator at discharge. At follow‐up, 13 patients (72.2%) had resolution of type 1 Brugada pattern ECG.

**Conclusion:**

COVID‐19‐associated Brugada pattern ECG seems relatively rare. Most patients had resolution of the ECG pattern once their symptoms have improved. Increased awareness and timely use of antipyretics is warranted in this population.

## Graphical abstract

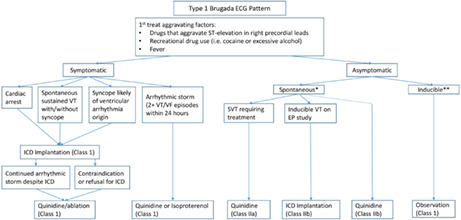

## 1 INTRODUCTION

In December 2019, the emergence of the coronavirus disease 2019 (COVID‐19), caused by the Severe Acute Respiratory Syndrome‐Coronavirus‐2 (SARS‐CoV‐2), led to the beginning of a global pandemic that has led to almost 2 million global deaths as of September 2021 (Elrobaa & New, 2021). The main clinical manifestation of COVID‐19 involves the pulmonary system, including respiratory tract symptoms such as fever, cough, and fatigue with complications ranging from mild flu‐like illness to acute respiratory distress syndrome (Johnson et al., 2020). However, emerging data in the early phases of the pandemic identified serious cardiovascular complications in COVID‐19 patients (Bansal, 2020; Johnson et al., 2020), which include cardiac arrhythmias, myocarditis, pericarditis, acute coronary syndrome, heart failure, cardiogenic shock, and cardiac arrest (Bader et al., 2021; Dherange et al., 2020; Nishiga et al., 2020). Currently, there are two proposed mechanisms that explain the development of cardiac complications in COVID‐19: First, SARS‐CoV‐2 has a binding affinity for the ACE‐2 receptor in myocardial cells and thus has elevated risk for direct cellular toxicity by entry and replication (Centurión et al., 2021). The second proposed mechanism relates to the heightened release of pro‐inflammatory cytokines through activation of the innate and adaptive immune system, often leading to a cytokine storm, which is a clinical entity, characterized by high and unrelenting fevers, often with temperatures >39.4**°**C (Centurión et al., 2021). Finally, the severity of these complications was further exacerbated in those patients with pre‐existing cardiovascular disease risk factors, such as hypertension, diabetes mellitus, and obesity (Sabatino et al., 2020).

Of the previously listed cardiac manifestations of COVID‐19, several studies have identified arrhythmias as one of the more common presenting complications. One study found 17% of COVID patients presented with an arrhythmia while a systematic review showed that up to 20% of hospitalized COVID‐19 patients developed an arrhythmia (Liao et al., 2020; Wang, Hu, et al., 2020). Among the spectrum of cardiac arrhythmias and ECG changes, atrial tachyarrhythmias, atrioventricular conduction blocks, ST‐T changes, and malignant arrhythmias have been highlighted as some of the more common manifestations (Li et al., 2020; Turagam et al., 2020; Wang, Wang, et al., 2020). More importantly, the development of arrhythmia during a hospitalization for COVID‐19 confers an increased mortality risk (Mountantonakis et al., 2021; Turagam et al., 2020). The hypercytokinemic state of COVID‐19's has been associated with deadly arrhythmias (Singh & Desai, 2020), though the exact mechanism is not fully understood. However, one such possible cause may be linked to underlying Brugada syndrome.

A current review of literature offers multiple case reports of the emergence of inducible type 1 Brugada ECG pattern in patients hospitalized with COVID‐19; however, due to its rare incidence there are no studies dedicated to its appearance. The purpose of this systematic review is to summarize published case reports of patients diagnosed with COVID‐19 infection and Brugada pattern ECG among patients without a prior diagnosis of Brugada.

## 2 METHODS

### 2.1 Eligibility criteria

Case reports that identified patients who had a confirmed COVID‐19 diagnosis by either PCR or other molecular amplification tests. Patients must have had an ECG identified Brugada pattern documented as well. Exclusion criteria included those with a prior known history of Brugada syndrome.

### 2.2 Search strategy and selection criteria

The reporting of this systematic review followed the standards of the Preferred Reporting Items for Systematic Review and Meta‐Analysis (PRISMA) Statement (Page et al., 2021). Two researchers independently performed the literature search, extracted the data, and assessed for study quality. This study protocol was submitted to the PROSPERO registration as well.

We searched PubMed, Embase, and Scopus using “COVID”, “Brugada”, and “Coronavirus” as keywords. The following data were extracted from included studies: baseline demographics past medical history, clinical presentation, diagnostic studies performed, medications received and clinical outcome for the patient, including the insertion of a cardioverter defibrillator. All patients had Brugada pattern type 1 based on HRS expert consensus diagnostic criteria. A synopsis of the data is provided in Table 1 below.

**TABLE 1:** Summary of patients' clinical characteristics and course.

Study	Age	Sex	Race	Presenting symptom	Medical history	Additional diagnostic studies	Significant test findings	Pharmacotherapy management	Result
Pasqualetto, 2020	57	M	Unknown	Shortness of breath Chest pain	Non‐Hodgkin lymphoma	Chest x‐ray	Bilateral consolidations on chest x‐ray CRP elevated to 122.5	Lopinavir/ritonavir Hydroxychloroquine	Resolution of Brugada
Boncoraglio, 2021	41	M	Unknown	Fever Nausea Myalgia Vomiting	Autism	CT chest CT abdomen/pelvis Echocardiography	CT A/P with pyelic distention CRP elevated to 119 Negative echo	Antipyretics Ceftriaxone	Resolution of Brugada
Kim, 2020	43	M	Filipino	Cough Fever Myalgia Shortness of breath	None	Chest x‐ray	Multifocal pneumonia	Acetaminophen Remdesivir	Resolution of Brugada
Vidovich, 2020	61	M	Hispanic	Chest Pain Fever Shortness of breath	Hepatitis C Obesity	Catheterization Chest x‐ray Echocardiography	Bilateral pneumonia Negative echocardiography	Adenosine	Resolution of Brugada
Choi, 2020	19	M	Hispanic	Cough Chest pain Fever Shortness of breath	Obesity Obstructive sleep apnea	Chest x‐ray	Bilateral pneumonia CRP > 300	Hydroxychloroquine	Resolution of Brugada
Guragai, 2019	46	M	Unknown	Productive Cough Chills Fever Headache Myalgia	None	CT chest	Bilateral pneumonia	Hydroxychloroquine antipyretics	No resolution / unclear follow‐up
De Nigris, 2021	7	M	Unknown	Abdominal pain Fever Vomiting	None	Echocardiography	BNP 763 CRP elevated to 53 Echocardiography: EF 50% w/ moderate pericardial effusion	IVIG Methylprednisolone	Resolution of Brugada
Pasquetto, 2020	52	M	Unknown	Fever Syncope	None	CT chest	Bilateral pneumonia CRP elevated to 160	Acetaminophen Augmentin	Resolution of Brugada wearable cardiac defibrillator
Mahadevaiah, 2020	40	M	Unknown	Chest pain Fever	None	Echocardiography	Negative echo	Antipyretics	Resolution of Brugada
Koo, 2020	57	M	Unknown	Chest pain Cough Fever	None	Chest x‐ray	Pneumonia	Supplemental oxygen	Resolution of Brugada
Chang, 2020	49	M	Bangladeshi	Fever Syncope	None	Catheterization Echocardiography	Negative cath Negative echo	Acetaminophen	Resolution of Brugada implantable cardiac defibrillator
Tsimploulis, 2020	53	M	Hispanic	Fever Shortness of breath	None	Chest x‐ray	Bilateral pneumonia normal BNP elevated procalcitonin	Azithromycin IV acetaminophen Hydroxychloroquine	Resolution of Brugada s/p CPR, passed away later.
Adedeji, 2021	44	M	Hispanic	Abdominal pain Diarrhea Fatigue Headache Myalgias Productive cough Shortness of breath	None	Chest x‐ray	Moderate pulmonary interstitial and alveolar edema Cardiomegaly	Acetaminophen Benzonatate	Resolution of Brugada
Lugenbiel, 2020	53	F	Unknown	Cough Diarrhea Fatigue Fever Myalgia	None	CT chest	Bilateral pulmonary ground‐glass opacities	Caspofungin Ceftriaxone Hydroxychloroquine	Unknown
van de Poll, 2020	58	M	Unknown	Abdominal pain Chest pain Cough Fever Shortness of breath	AVNRT s/p ablation	Chest x‐ray	Consolidation in lower lobe CRP elevated to 64	Antipyretics Supplemental oxygen	Resolution of Brugada pattern
van de Poll, 2020	40	M	Unknown	Chills Cough Fever Syncope	None	Chest x‐ray	Left and possible right consolidation CRP elevated to 100	Antipyretics	Resolution of Brugada pattern
Chawla, 2021	72	F	Unknown	Chest pain Cough Fever	COPD	Chest x‐ray	Normal coronaries on cath	Antipyretics Dexamethasone Remdesivir	Wearable cardiac defibrillator
Dommaraju, 2021	57	M	Unknown	Cardiac arrest	None	Catheterization Chest x‐ray Echocardiography	TTE: LVEF 25% with severe diffuse hypokinesia of LV Normal coronaries on cath	Vasopressors Temporary LVAD	Unknown

## 3 RESULTS

**FIGURE 1:**
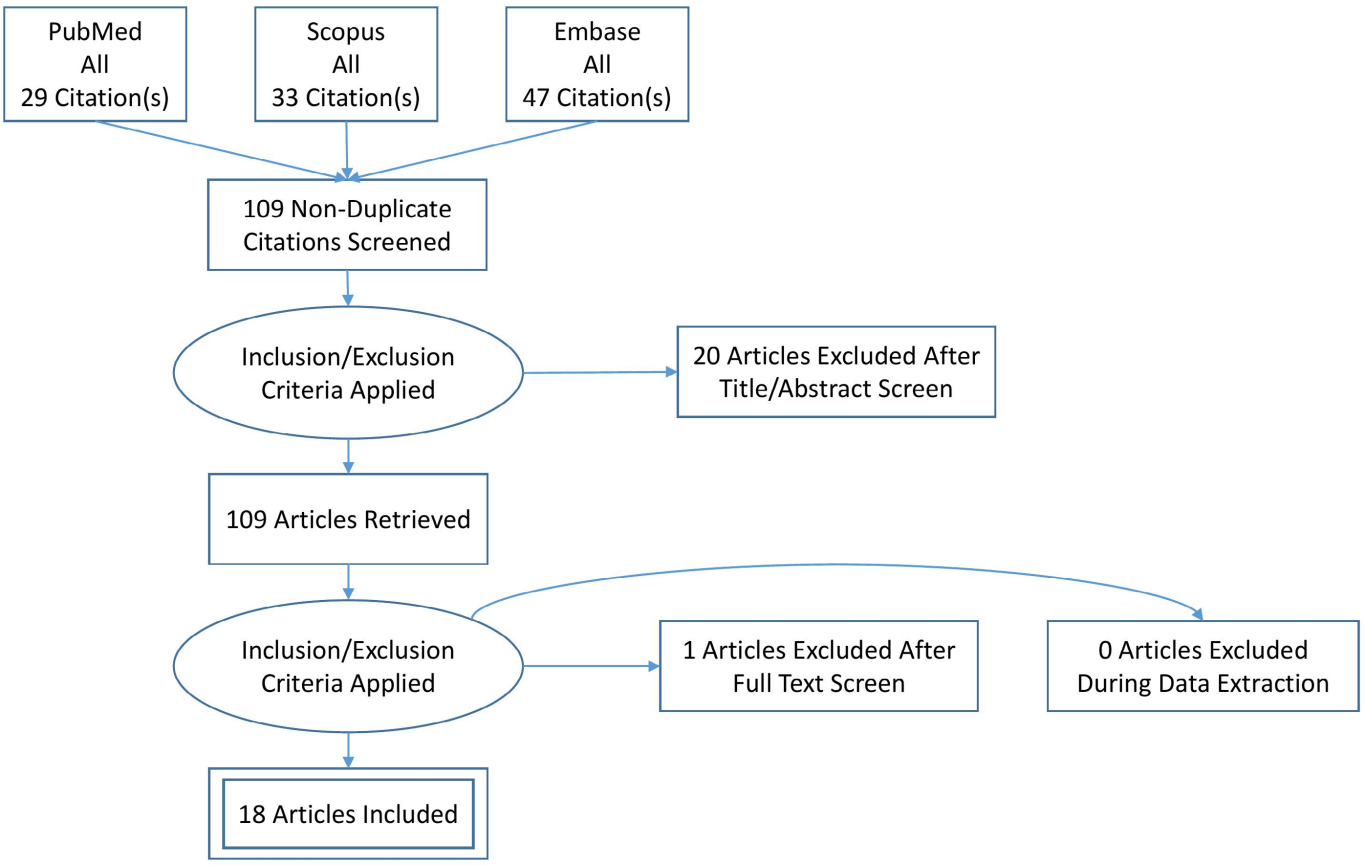
PRISMA flow diagram of included studies.

Overall, 109 studies were captured in our initial search strategy and is illustrated in the recommended PRISMA flow diagram (Figure 1). After the screening process, 19 studies were identified. One study was excluded after full‐text analysis as the patient had a known Brugada syndrome diagnosis.

The mean age was 47.1 years with 11.1% being women. No patients had prior confirmed diagnosis of Brugada syndrome. The most common presenting clinical symptoms were fever (83.3%), chest pain (38.8%), shortness of breath (38.8%), and syncope (16.6%). All 18 patients presented with type 1 Brugada pattern ECG. Four patients (22.2%) underwent left heart catheterization, and none demonstrated the presence of obstructive coronary disease. The most common reported therapies included antipyretics (55.5%), hydroxychloroquine (27.7%), and antibiotics (16.6%). One patient (5.5%) died during hospitalization. Three patients (16.6%) who presented with syncope received either an implantable cardioverter defibrillator or wearable cardioverter defibrillator at discharge. At follow‐up, 13 patients (72.2%) had resolution of type 1 Brugada pattern ECG.

## 4 DISCUSSION

The emergence and persistence of the COVID‐19 pandemic demonstrates the importance of recognizing how this disease interacts with other medical conditions, and how this may change management. However, in this systematic review, for those with only Brugada pattern on ECG, rather than Brugada Syndrome, the fever did not seem to have any proclivity toward an arrhythmic arrest and had a 72% resolution of ECG changes upon recovery. Additionally, for those few that had ischemic evaluation through left heart catheterization, no cases demonstrated any significant coronary obstruction. This would suggest that COVID‐19 is an inducible factor for reversible Brugada‐pattern on ECG that, without symptoms, does not necessitate device implantation (Chang et al., 2020; Vidovich, 2020) similar to those induced by fever or medication but not present at baseline. Of those that presented with syncope, all eventually were discharged with an implantable cardioverter defibrillator or wearable cardioverter defibrillator, much in line with current expert consensus and recommendations for Brugada Syndrome management (Al‐Khatib et al., 2018; Priori et al., 2013). Thus, those with presentation of syncope or cardiac arrest should still prompt evaluation for ICD implantation as per current recommendations (Al‐Khatib et al., 2018; Priori et al., 2013), since specific medium‐ to long‐term outcome data are not currently known with the data available with regards to COVID‐19 related Brugada pattern and cannot suggest otherwise.

It cannot be determined for certain what induced the Brugada ECG pattern, however, it is most likely related to fever. Current literature demonstrates a well‐known connection between fever and Brugada Syndrome and the need for antipyretic treatment (Adler et al., 2013; Michowitz et al., 2018; Priori et al., 2013): increases in body temperature have been proven to cause sodium channel deactivation, and thus increased phase 2 re‐entry ventricular arrhythmias and potentially sudden death in Brugada Syndrome (Antzelevitch & Brugada, 2002; Keller et al., 2005; Sorgente et al., 2020). At this point in time, there is no data regarding the direct effect of COVID‐19 upon sodium channels that could lead to any physiologic effect upon those with Brugada Syndrome, nor medium‐ or long‐term outcome data surrounding the two. COVID‐19 has also been postulated to create ST elevation through microthrombi and myocarditis without obstructive coronary artery disease (Sorgente et al., 2020) in those who end up with an angiographically normal coronary angiography, as also demonstrated in our review of cases, though no studies have been dedicated to investigating this hypothesis yet. However, emerging data suggest the possibility of selective ion channel dysfunction in the setting of immunologic/inflammatory insult (Lazzerini et al., 2018), which may warrant further investigation specifically in the connection between the COVID‐19 hypercytokinemic state and its effect on ion channels.

**FIGURE 2:**
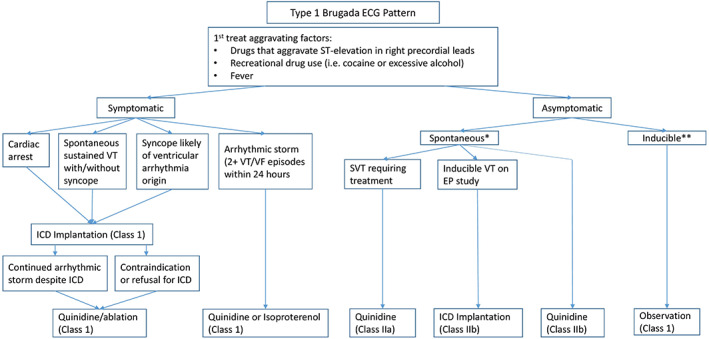
A proposed treatment algorithm for patients presenting with Type 1 Brugada‐pattern ECG. *Spontaneous Brugada pattern is described as a Type 1 Brugada ECG pattern that does not resolve with resolution of aggravating factors. ** Inducible Brugada pattern is described as a Type 1 Brugada ECG pattern that resolves with removal of aggravating factors.

Despite data regarding the known effects of aggravating factors for Brugada‐pattern ECG, the various forms and labels of Brugada classification may cause confusion with regards to management. Examples include inducible versus spontaneous, symptomatic versus asymptomatic, Brugada‐pattern ECG versus Brugada Syndrome. Thus, we propose the below algorithm to help guide management in patient's presenting with a new Brugada‐ECG pattern to merge the algorithm and recommendations provided by Alkhatib et al with the HRS expert consensus by Priori et al, adding an algorithm prior to the diagnosis of Brugada syndrome and creating an arm for inducible Brugada pattern (Figure 2).

There are several limitations to this review. The major limitation of this study is the retrospective collection of case reports and small case series of a rare finding (Brugada pattern) in an already limited pool of patients of those admitted with COVID‐19, currently yielding a limited sample pool with the usual associated biases of such analysis. Work‐up and pharmacological therapy provided was limited by secondary account in case reports, and thus a standardized review of studies could not be performed. Similarly, race/ethnicity was not reported in a majority of the case reports, preventing complete evaluation of the baseline population.

Despite the limited data pool available, the baseline data do seem to reflect an approximate sample population based on current knowledge of Brugada Syndrome, with a known proclivity to male dominance (our measured 9:1 ratio) (Benito et al., 2008), and similar incident age of diagnosis (our measured 47 years, compared to average age of 41 years) (Priori et al., 2002).

## 5 CONCLUSION

As the pandemic continues, there will be more patients with COVID‐19‐associated Brugada pattern, which will allow for further accuracy in characterization of such presentations. The emergence of Brugada pattern on ECG during COVID‐19 requires careful monitoring to ensure no development of arrhythmic complications through antipyretic use and continuous ECG monitoring similar to other causes of fevers with such ECG pattern. The ECG pattern reverses in most cases with fever resolution so there does not appear to be a role for implantation of implantable cardioverter defibrillators. Future direction could be aimed toward investigating possible effects of COVID‐19 upon sodium channels in any, and the long‐term consequences of transient COVID19‐induced Brugada pattern ECG changes and/or Brugada syndrome.

## AUTHOR CONTRIBUTIONS

All persons who meet authorship criteria are listed as authors, and all authors certify that they have participated sufficiently in the work to take public responsibility for the content, including participation in the concept, design, analysis, writing, or revision of the manuscript. Furthermore, each author certifies that this material or similar material has not been and will not be submitted to or published in any other publication before its appearance in the Annals of Noninvasive Electrocardiology.

## FUNDING INFORMATION

This research did not receive any specific grant from funding agencies in the public, commercial, or not‐for‐profit sectors.

## CONFLICT OF INTEREST STATEMENT

All authors have no Conflict of Interest.

## ETHICAL APPROVAL

This research did not contain any studies involving animal or human participants, nor did it take place on any private or protected areas. The declaration of Helsinki was adequately addressed and no specific permissions were required for corresponding locations.

## Data Availability

Data sharing is not applicable to this article as no new data were created or analyzed in this study.
